# GFZ Wireless Seismic Array (GFZ-WISE), a Wireless Mesh Network of Seismic Sensors: New Perspectives for Seismic Noise Array Investigations and Site Monitoring

**DOI:** 10.3390/s100403280

**Published:** 2010-04-01

**Authors:** Matteo Picozzi, Claus Milkereit, Stefano Parolai, Karl-Heinz Jaeckel, Ingo Veit, Joachim Fischer, Jochen Zschau

**Affiliations:** 1 Helmholtz-Zentrum Potsdam Deutsches GeoForschungsZentrum, Telegrafenberg, 14473 Potsdam, Germany; E-Mails: online@gfz-potsdam.de (C.M.); jaeckl@gfz-potsdam.de (K.-H.J.); parolai@gfz-potsdam.de (S.P.); zschau@gfz-potsdam.de (J.Z.); veit@gfz-potsdam.de (I.V.); 2 Department of Informatics, Humboldt University (HU) Berlin, Germany; E-Mail: fischer@informatik.hu-berlin.de

**Keywords:** sensor network, seismic array, wireless mesh network, site effects, MEMS, earthquake risk

## Abstract

Over the last few years, the analysis of seismic noise recorded by two dimensional arrays has been confirmed to be capable of deriving the subsoil shear-wave velocity structure down to several hundred meters depth. In fact, using just a few minutes of seismic noise recordings and combining this with the well known horizontal-to-vertical method, it has also been shown that it is possible to investigate the average one dimensional velocity structure below an array of stations in urban areas with a sufficient resolution to depths that would be prohibitive with active source array surveys, while in addition reducing the number of boreholes required to be drilled for site-effect analysis. However, the high cost of standard seismological instrumentation limits the number of sensors generally available for two-dimensional array measurements (*i.e.*, of the order of 10), limiting the resolution in the estimated shear-wave velocity profiles. Therefore, new themes in site-effect estimation research by two-dimensional arrays involve the development and application of low-cost instrumentation, which potentially allows the performance of dense-array measurements, and the development of dedicated signal-analysis procedures for rapid and robust estimation of shear-wave velocity profiles. In this work, we present novel low-cost wireless instrumentation for dense two-dimensional ambient seismic noise array measurements that allows the real–time analysis of the surface-wavefield and the rapid estimation of the local shear-wave velocity structure for site response studies. We first introduce the general philosophy of the new system, as well as the hardware and software that forms the novel instrument, which we have tested in laboratory and field studies.

## 1. Introduction

Recent strong earthquakes worldwide (e.g., Michoacán, Loma Prieta, Kobe, Izmit) have provided clear evidence that the damage that results at a site is not merely a function of the energy released from the earthquake source. In fact, the level of damage and devastation in urbanized area might follow a very complex pattern also related to a phenomenon called ‘site effect’ that is due to those variations of geological and geotechnical conditions at shallow depth (*i.e.*, essentially the shear-wave velocities of soft-sediments and of the bedrock) that significantly affect the seismic shaking at the surface.

For this reason, knowledge of the local near-surface shear wave (S-wave) velocity profile is critical for estimating the damage and loss potential patterns from future earthquakes, as it plays the main role in effects such as ground-motion amplification, landslides or liquefaction. The evaluation of site-effects is therefore one of the key components for mitigating the effects of earthquake disasters. Hence, to mitigate the risk associated with the recurrence of earthquakes, a number of procedures suitable for mapping the mechanical properties of the ground (*i.e.*, the S-wave velocity profile) near the surface (*i.e.*, typically between the first thirty and one hundred meters) and to account for site effects in the levels of ground shaking expected have been provided. The application of these procedures is defined seismic microzonation. However, the mitigation of seismic risk in urban area requires the estimation of the S-wave velocity profile, and thus of the earthquake ground motion amplification, over large areas. This can be accomplished only if methods suitable for the particular urban environment are developed and applied. Conventional seismic methods (reflection, refraction, cross-hole, down-hole, *etc.*) require artificial sources or the drilling of boreholes, which are both expensive, effective for restricted investigation depth only (a few tens of meters), and difficult or impossible to implement in urban or environmentally sensitive areas. For this reasons, in the last decades the analysis of the very small amplitudes Earth’s surface vibrations (defined ‘seismic-noise’ or ‘microtremors’, and having displacement generally included in the range 10^−4^ 10^−2^ mm) produced by natural or anthropic sources, and that can be recorded with good lateral coverage and at reasonable costs, captured the interest of the geophysicist community. In particular, since the pioneering work of [1], two-dimensional (2D) seismic arrays have been used at small scales (*i.e.*, maximum aperture of the array is of the order of tens to hundreds of meters) for the characterization of surface-wave propagation, and the extraction of information about the shallow subsoil structure (*i.e.*, the estimation of the local S-wave velocity profile).

Over the last few years, due to the focus of seismologists and engineers on estimating the amplification of earthquake ground motion as a function of local geology, and the improvements in the quality and computing power of instrumentation, the analysis of seismic noise recorded by 2D arrays has been confirmed to be particularly successful in deriving the subsoil S-wave structure (e.g., [2–6]). Using just a few minutes of seismic noise recordings and combining this with the well-know horizontal-to-vertical spectral ratio (H/V) method, it has also been shown that it is possible to investigate the average one-dimensional (1D) velocity structure below an array of stations in urban areas with a sufficient resolution to depths of also few hundreds of meters that would be prohibitive with active source array surveys, and while also reducing the number of boreholes required to be drilled for site-effect analysis. Comparisons of the theoretical site response from 2D arrays with empirical ones from earthquake recordings at seismic stations indicate that 2D array seismic noise methods allow the estimation of the most relevant and reliable information about the local S-wave structure for site response.

Amongst these studies in recent years, the use of micro-seismic arrays for seismic noise recordings have proved to provide vital information for rapid and cost-effective microzonations of urban areas (among the others [7–9]). Until now, a crucial point limiting the applicability of these array measurements in urban area is that expensive, heavy and stand alone stations to be deployed in the field are required, especially when techniques aimed at resolving the subsoil three dimensional, 3D, structure [10] are applied. This of course limits the number of stations that can be used simultaneously, therefore limiting the success of the experiments. Techniques have been developed to overstep this drawback, but very often they require both strong assumptions on the wavefield’s nature and the usage of array geometries that are difficult to implement in urban areas. Moreover, standard arrays are deployed using stand-alone stations that do not allow a fast check of the data quality in the field, limiting the potential application of the data acquisition.

A promising solution to these issues is provided by the rapid improvement in telemetry and computer technology, which is literally driving a revolution in seismology and earthquake engineering. The earliest application of wireless communication technology started in the late 90s [11], when wireless sensors were connected together with embedded PC for structural monitoring purposes. These earlier applications first showed that real-time processing of data can be performed locally, and that wireless monitoring systems are feasible, reliable and cost effective. Over the last few years, prototype structural wireless monitoring systems have been validated by tests performed on bridges and other structures [12], where they have been found to be highly cost-competitive, completely autonomous and very reliable alternatives to traditional wired systems.

Recently, Ohrnberger *et al*. [13] firstly proposed to use a wireless mobile ad-hoc network of standard seismological stations equipped with high sensitivity, but also highly expensive, Earth Data digitizers for site-effect estimate applications. The system allows for the retrieval of data in real time, and thus, to undertake preliminary in-field data processing. To overcome the resolution problem posed by a reduced number of stations available, the authors proposed to repeat the measurements using consecutive arrays with different sizes.

At the present time, the *Helmholtz-Zentrum Potsdam Deutsches GeoForschungsZentrum* (GFZ-Potsdam) and the *Humboldt University of Berlin* (HU-Berlin) are developing an innovative, self-organizing wireless mesh information network made up of low-cost sensing units equipped with Micro Electro Mechanical Systems (MEMS) accelerometers, with the aim of setting up earthquake early warning systems for mega cities [14,15]. This innovative system, named the Self-Organizing Seismic Early Warning Information Network (SOSEWIN) was developed within the framework of the European projects SAFER (Seismic eArly warning For EuRope, http://www.saferproject.net) and EDIM (Earthquake Disaster Information systems for the Marmara Sea region, Turkey, http://www.cedim.de/EDIM.php), and a first test version has been deployed since July, 2008, in Istanbul, Turkey.

Taking advantage of the experience gained during the SAFER and EDIM projects, we developed a new, dedicated system for seismic arrays, named the GFZ WIreless SEismic array (GFZ-WISE) made up of a large number of low-cost Wireless Sensing Units (GFZ-WSU), which allow dense 2D seismic ambient-noise arrays to be deployed. We verified that the MEMS accelerometric sensors used by the SOSEWIN sensors do not have the sufficient resolution for seismic noise measurements and analysis. Therefore, for such a specific task the GFZ-WSUs are equipped with passive external geophones.

Innovatively, the GFZ-WISE system will create a self-organizing wireless mesh network that will be capable to flexibly adapt to broad range of users and unforeseen network development, as, for instance, if changes in the network configuration will occur for the increase of the sensor number, or decrease if some of them will fail. During seismic noise investigations, these arrays will allow the real-time retrieval, *via* the SeedLink protocol [16], and analysis of data for the rapid estimation of the local S-wave velocity structure.

In the framework of site monitoring activities, the GFZ-WISE might be exploited to continuously estimate the subsoil mechanical properties, for example of landslides. In particular, it could allow, through the joint analysis of the multi-parameter data (e.g., the S-wave velocity, ground motion, groundwater level variation, and rain gauge) and dedicated decision making algorithms, to detect and locate changes within the landslide, and also provide real time early warning information about the possible landslide activation after earthquakes or meteorological events. On the other hand, in the urban context the GFZ-WISE system could be used for monitoring the variation of the subsoil mechanical properties following the shaking of an earthquake and to study the soil-structure interaction effects. In this paper, we first describe the general philosophy of the GFZ-WISE, as well as the hardware and software characteristics of the WSUs. Then we report on the laboratory and field tests performed, in particular the field experiment using the GFZ-WISE within the Alfred Einstein Science Park, Potsdam, Germany.

## 2. GFZ-Wireless Sensing Unit (GFZ-WSU): Hardware And Communications

The development of the GFZ-WISE system has focused on two points. The first is the development of a low-cost wireless sensing unit (*i.e.*, the hardware), while the second concerns the creation of a self-organizing wireless mesh network (*i.e.*, the software). Both of these steps have been made possible by taking advantage of the experience gained during the development of the Self Organizing Seismic Early Warning Information Network developed during the SAFER and EDIM projects [14].

**Figure 1.**
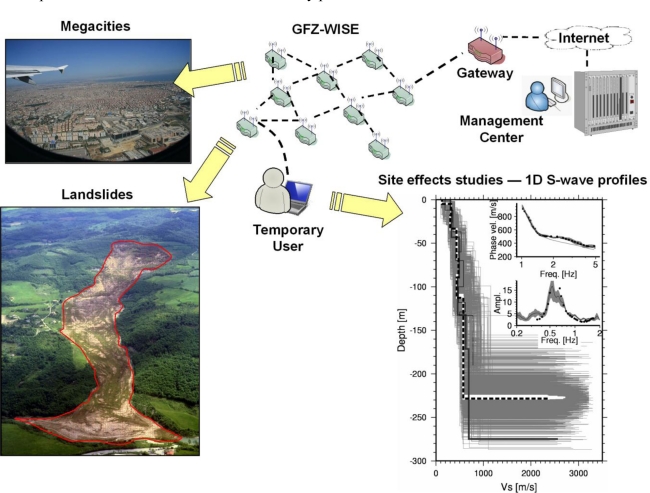
Illustration of the proposed applications of the GFZ-WISE. The network consists of wireless stations that for long-term monitoring purposes can be linked to a central processing centre, while during temporary surveys transmit data in real-time to an external user. This allows the user to perform real-time inversion analysis of dispersion and H/V spectral ratio curves for the S-wave velocity profile estimation.

The GFZ-Wireless Sensing Unit (GFZ-WSU) costs less than one tenth of a standard instrument, and is able to collect, store and undertake preliminary analysis of data when only deriving basic parameters (e.g., the H/V curve of a site) is of concern. Figure 1 shows the general philosophy of the GFZ-WISE system. Each GFZ-WSU node records the ground motion and transmits the data by the SeedLink protocol [16]. Through the Optimized Link State Routing (OLSR) protocol [17], these novel sensors can create a dense, self-organizing and decentralized seismic monitoring network. Raw data and computed parameters can be communicated to an external laptop running the SeisComP software [16] when the laptop is connected to any node that belongs to the network, allowing real-time analysis of the seismic data. In the following, the main characteristics of the software ensuring the system functionality (*i.e.*, SeedLink, SeisComP, and OLSR) are reported.

The self-organizing character allows GFZ-WISE to automatically adapt to changes in the network configuration, and guarantees the functionality of the network even when some of the sensing units malfunction, or cannot be seen by the external user. These characteristics make the system particularly suitable for application within mega cities and the monitoring of areas at risk to landslides. In particular, within the context of monitoring applications, special nodes equipped with additional communications hardware, e.g., Internet connection, satellite phones, VSAT, *etc*., may serve as entities able to communicate data and parameters to outside the network, such as to a disaster management center. On the other hand, the system is defined ‘decentralized’ because the data and the estimated parameters will not be transmitted to a unique user, or management center, but will be also available at every node of the network. Hence, during monitoring activities the decentralized property will allow, together with dedicated decision making algorithms, to perform a cooperative analysis of the data for early warning purposes.

### 2.1. Hardware

**Figure 2.**
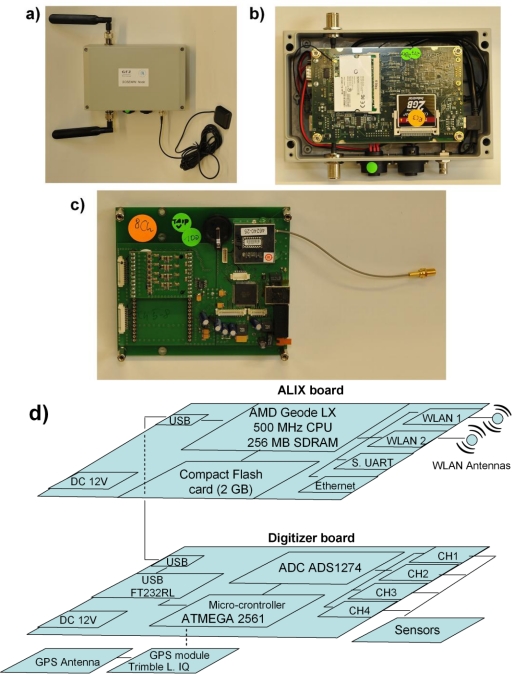
The prototype GFZ Wireless Sensing Unit (GFZ-WSU). (a) The complete unit. (b) The WRAP board. (c) The ADC board. (d) A schematic overview of the architecture of the GFZ-WSU. Technical details of the various components are listed in Tables 1, 2, and 3.

The GFZ-WSU consists of three main hardware parts: the digitizer board, the Wireless Router Applications Platform (WRAP, *i.e.*, the PC Engines ALIX system board), and the sensors. All components are bought off-the-shelf, with the exception of the digitizer printed circuit board, which has been developed within GFZ Potsdam. This reduces the cost of the GFZ-WSU, leading to them being much less expensive than standard seismometers (about 700 Euro per unit). Figure 2 provides a view and schematic overview of the architecture of a GFZ-WSU, with some technical details listed in Tables 1, 2, and 3. All boards are installed in waterproof outdoor metal cases of reduced dimension and weight (Figure 2a, Table 1). Omni-directional dual-band antennas with a gain of 5 dB are mounted with opposite vertical polarization. The amount of power required by a WSU when all operational activities are fulfilled (recording and real-time communication of data) has been experimentally measured to be about 4.5 W.

The Analog-to-Digital Converter (ADC) board (Figure 2c, Table 3) was designed as a low-cost solution, but still considers special seismic requirements as there are high resolution, anti-alias filtering, exact time marks and good time stability included in its design. The board is equipped with a 4-Channel ADC (ADS1274) or 8-Channel ADC (ADS1278). These ADCs have a resolution of 24 bits (effectively 19 bits in low-power mode, and 20 bits in high-resolution mode), with the sampling rate selectable from 100 to 400 samples per second (sps), although at present 100 sps is being used. The cut-off frequency of the digital anti-alias filter is close half of the used sampling rate.

A GPS unit (Trimble Lassen iQ) provides time and geographical coordinates (Figure 2c). Every second it sends a PPS (pulse per second) to the ATMEGA-2561 micro-controller. This PPS is used to mark the first sample of a second. The GPS time and the position are also transferred to the digitizer board *via* TAIP strings. The complete power consumption of the board is 540 mW and 720 mW for the low-power and high-resolution mode, respectively, including GPS module and antenna.

The ALIX board ALIX.3D2 [18] has the three roles of analysis, communication, and storage of data (Figure 2b, Table 2). It is made up of an embedded PC (AMD LX800 500 MHz CPU, 256 MB RAM) that uses a Compact Flash card (currently 2 GBytes, but easily increased) as a hard disk. Importantly, the ALIX board is equipped with two positions for WLAN Mini PCI cards (*i.e.*, Routerboard R52 wireless 802.11a/b/g, 2.4 and 5 GHz combo cards that use the Atheros AR5414 chipset, [19]), a power supply plug, a serial port USB and 100 MBit/s Ethernet. Power consumption is from 3 to 5 W at 12 V DC. Figure 2d illustrates schematically how the different components (e.g., WRAP, ADC, *etc*.) of a GFZ-WSU are organized.

The GFZ-WSU is designed to accommodate different kinds of sensors, since the ADC board has four or eight channels and is designed to host contemporarily different modules, thus, leading to the use of instruments considered to be suitable for monitoring different parameters. In particular, for seismic noise measurements, a preamplifier board is added (Table 1), and standard 3D SM-6/B 4.5 Hz external geophones, as well as any other passive sensor, can be connected to the instrument.

Furthermore, accelerometric sensors based on MEMS (Micro Electro Mechanical Systems), originally designed to serve as controllers for air bag safety units, but which have also been successfully incorporated into various seismic networks [22], as well as for field acquisition by the exploration sector [23] can be incorporated into the GFZ-WSU and arranged to provide three component data. Actually, the MEMS units tested at the moment have a measurement range of +/− 1.7 g, with a bandwidth of 25 Hz and a noise-level of 0.2 mg [14]. Picozzi *et al*. [24] successfully exploited a version of the GFZ-WSU instrumented with MEMS units for the monitoring of some strategic infrastructures during the Earthquake Task Force mission following the recent magnitude (Mw) 6.3 Central Italy Earthquake of the 6th April 2009. However, it is worth noting that the resolution of the MEMS sensors so-far incorporated into the GFZ-WSU is not sufficient for seismic noise measurements and analysis.

At the present, preliminary tests (not shown here) have been performed with temperature sensors, pressure sensors, and microphones (for low frequency audio signals) with analog output signals up to ±2.5 V directly to one of the input channels in the ADC board not connected to a ground-motion recording instrument.

For special monitoring applications (e.g., in the case of landslides) where access to main power is limited, the necessary energy can be provided by solar panels. Tests carried out at the GFZ-Potsdam and observations from the test-bed seismic early-warning network in Istanbul [14], where some wireless stations are equipped with a buffer battery and connected to a solar panel through a solar controller, indicate that a 60 W solar panel and a 40 Ah battery can deliver the necessary energy for nearly continuous operating status. In these cases, the one of the channels of the ADC board can be used for the battery voltage surveillance.

### 2.2. Software

The main software operating on the WSU currently consists of the following:

OpenWRT: The operating system for the WRAP boards [20] with Linux kernel 2.6.22 [21]. OpenWRT is an open-source freely available and highly configurable distribution. By default, it contains only the minimum that is required to run Linux, so it can also run on very size-limited systems. Moreover, it provides an environment for building your own Linux distribution for several platforms, including our x86er target platform for the WRAP boards.

Data-provider: The program that handles the data streams from the digitizer board, and then archives them *via* SeedLink (SeisComP).

SeedLink (SeisComP): The Seismological Communication Processor (SeisComP) is an open-source software package and concept for near real-time seismic data distribution, (http://geofon.gfz-potsdam.de/geofon//SeisComP/seedlink.html) developed by the GFZ for a networked seismographic system. In particular, in a seismic network SeisComP is responsible of the following tasks: data acquisition, data recording, monitoring and controlling, real-time communication, user access, and automatic (near-)real-time data processing (quality control, event detection and location). The SeedLink program is part of SeisComP, and is the system devoted to the near real-time seismic data distribution, that is, a server protocol based on *Transmission Control Protocol* (TCP) [16]. In particular, *via* SeedLink the data are sent in the form of 512-byte Mini-SEED packets with a 8-byte SeedLink header. The header contains the packet sequence number, which allows the unit to resume transmission where it left off (*i.e.*, the recovering of the connection in the event of network errors, and the support of non-permanent connections in “dialup mode”). It has client-server architecture and is capable of many tasks (data acquisition, data recording, monitoring and controlling, real-time communications, user access, near-real-time data processing). In the WSU, the SeedLink server stores the data in a ring buffer of configurable size on the Compact Flash card. The data in the ring buffer will be kept for the order of 20 days. If more storage is found to be necessary, then it is simply a case of using a larger Compact Flash card. Actually, SeedLink is the only component of SeisComP used by the GFZ-WISE. Nevertheless, in order to retrieve the data from the system, at the moment every user must install SeisComP on its computer.

Optimized Link State Routing [17]: OLSR is a table-driven pro-active routing protocol currently chosen for the wireless mesh network (http://www.olsr.org). As a proactive protocol it periodically assesses and maintains the network topology by flooding information about its direct neighborhood throughout the whole network. OLSR has proven that it is capable of operating with hundreds of nodes, and it is also widely accepted by several mesh networking communities, *i.e.*, Freifunk (http://www.freifunk.net) and the Funkfeuer (http://www.funkfeuer.at) projects.

### 2.3. Communication

The communication of seismic data and information amongst the GFZ-WSU is based on the routing concept. The term routing refers to the procedure of selecting within a network the paths along which data can be sent from a source to a sink. Routing activities within a wireless network are made more complicated by the fact that all nodes act contemporarily as sources, sink and routers of data. GFZ-WSUs rely on the OLSR (Optimized Link State Routing) protocols as the routing strategy (see above for the OLSR description), and are designed to form a self-organizing ad-hoc wireless mesh network (WMN). The use of WMN protocols allows a network of WSUs to continuously adapt to changing circumstances (addition or removal of nodes, interference in communications, loss of sensors due to unforeseen malfunctioning *etc*.) in order to maintain optimal communications [14]. The main advantages of WMN for seismic noise array surveys in urban context are (1) the system is free from cable usage, thus, allowing improved array geometries, and azimuthal coverage, (2) in the case of large arrays, data also can be transferred to a user by multi-hop communications from those instruments that are not in light-of-sight with the user itself (remote stations).

The OLSR that the GFZ-WISE employs is a *proactive* routing protocol, where every node has a map of the complete network topology, allowing data to be immediately sent along the optimal path towards the users or the gateways. This leads to each node having a routing table that describes the most efficient way to reach each other node. It makes use of advanced metrics, *i.e.*, measurement methods, for the evaluation of a multi-hop path within the network.

**Figure 3.**
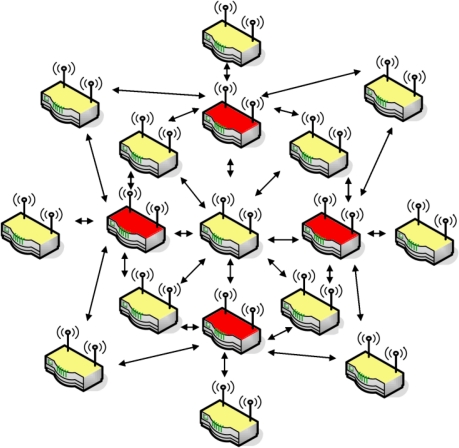
MultiPoint Relays protocol for communication within a dense array, *i.e.*, how messages from a central node are distributed throughout a cluster. In practice, only a subset of the central node’s neighbors (red) need to rebroadcast the message.

In order to make the transmission of messages as efficient as possible, particularly in limiting duplicate transmissions, a MultiPoint Relays (MPR) communication schema is adopted. This approach is important, especially when the network is done for monitoring purposes. Roughly speaking, each unit periodically broadcasts “Hello” messages to its direct neighborhood. These messages include the list of known neighbors, combined with the status of the quality of the connection to them. By knowing its two K-hop neighborhood, every node independently chooses a subset of the one K-hop neighborhood by which the complete two K-hop neighborhood is reachable. This results in certain nodes being designated as a MPR, which allows for a reduction in transmissions when flooding the network as only the MPRs need to rebroadcast a message to reach the complete 2 K-hop neighborhood (Figure 3). Every node also announces its chosen MPR, so that each node knows if it is a MPR or not. Nodes selected as MPR regularly flood the network with topology control (TC) messages at a defined interval (less frequently than “Hello” messages). These messages contain the link states of the nodes that selected this node as a MPR (the MPR selectors). By receiving these messages, a node therefore has enough information to locally reproduce the complete topology of the network. This enables a node to compute optimal paths to all known destinations, which in OLSR is done using Dijkstra’s shortest path algorithm [25].

Within the GFZ-WISE, seismic data are transferred among stations using a multi-hops strategy by means of the SeedLink protocol (*i.e.*, by 512-byte Mini-SEED data packets), with a rate of up to 54 Mbps in both the 2.4 GHz and 5 GHz unlicensed bands. In the case of a low signal-to-noise ratio in the communications, the WLAN cards driver can automatically decrease the rate of transmission. Tests performed within the framework of seismic early-warning activities in Istanbul, Turkey, using instruments with the same software and a similar hardware configuration showed that WLAN communications between line-of-sight stations equipped with omni-directional antenna is possible until a distance of ca. 250 m within an urban context [14].

## 3. Laboratory Tests: GFZ-WSU *versus* Earth Data PR6-24

**Figure 4.**
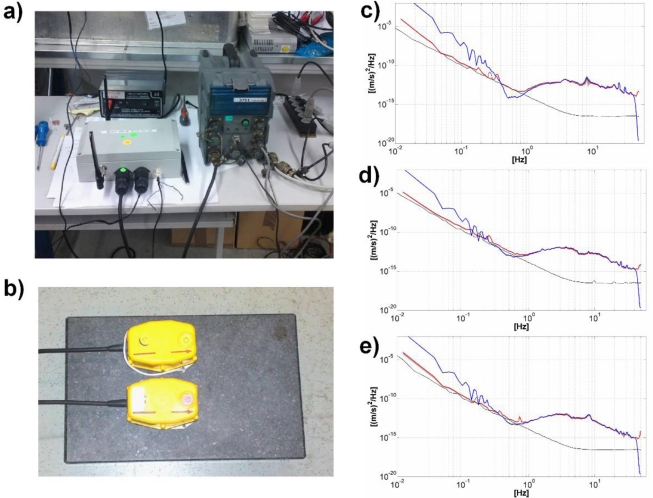
Results from the laboratory experiment. (a) GFZ-WSU and EDL digitizer. (b) 3D SM-PE-6/B 4.5 Hz external geophones deployed over a concrete slab. (c) Spectra from EDL (*blue*), WSU (*red*), WSU’s self-noise (*black*) for the vertical component of ground motion. (d) same as (c) but for the north-south component. (e) same as (c) but for the east-west component.

Preliminary tests of the GFZ-WSUs performance when connected to a standard 3D SM-PE-6/B 4.5 Hz external geophone consisted of comparing the signals recorded under laboratory conditions with contemporary ones acquired using a highly sensitive standard Earth Data PR6-24 (EDL), a 24 bit digitizer, and the same kind of sensor. The sensors for both stations are deployed on a concrete slab (Figure 4a,b), with the digitizer gain for each set to 10.

Figures 4c–e shows the spectra for the three component of motion for the two instruments. Additionally, we show also the spectra of the GFZ-WSU self-noise, which allows for an estimation of the frequency range over which signals can be reliably recorded [26,27]. Interestingly, under the noise conditions existing during the experiment, the GFZ-WSU’s spectra is in excellent agreement with the EDL’s ones down to 1Hz. Below this frequency, due to the lower sensitivity of the GFZ-WSU relative to the EDL digitizer, the seismic noise approaches the instrumental’s self-noise and the recorded signals represent only the self-noise itself.

It is worth pointing out that, being a function of the noise conditions, it is not possible to state a priori during field measurements which will be the lower frequency limit for recording reliable signals. Therefore, in order to define the frequency range where signals may be properly recorded, we consider it mandatory, also in the case when standard instrumentation is used, to compare experimental spectra together with the self-noise obtained for the specific combination digitizer-level to gain-sensor.

## 4. Field Tests of the GFZ-WISE

The GFZ-WISE is being developed with the primary goal of performing real-time analysis of data during seismic array measurements for the estimation of S-wave velocity profiles. Once the system is deployed in the field, the GFZ-WSUs create a WMN, allowing an operator with a laptop, where the SeisComP software is running, to retrieve in real-time data from all stations of the network. Therefore, signal analysis procedures can be performed from the user directly on the field. In the meantime, each station continuously stores the raw data in the ring buffer contained on the flashcard (*i.e.*, about 20 days worth on a 2 GB card).

### 4.1. Methodology of Surface-Wave Analysis

Consistent and reliable estimates of parameters related to site effects from seismic noise measurements are commonly retrieved from the analysis of the horizontal-to-vertical (H/V) spectral ratio curve [e.g., 28,29], and the Rayleigh wave dispersion curves (RWDC) (e.g., [3,5,7,30–33]).

Microtremors are highly variable and irregular assemblages of seismic waves (e.g., body waves, surface waves, and their related scattered and diffracted phases). Among them, surface waves (*i.e.*, Rayleigh and Love waves) are considered to be the dominant and the most coherent component of microtremors. Therefore, when a network (array) of vertical seismometers is used for microtremor recordings, information on the Rayleigh wave propagation in the medium can be extracted. The great interest that seismologists reserved for these waves is mainly justified by the relationship existing between their velocity and the subsoil structure, and in particular the S-wave velocity. Hence, considering that Rayleigh waves sample portions of the subsoil proportional to their wavelengths, and that their phase velocities are strongly conditioned by the S-wave velocities of the layers sampled, they are used to deduce information on the subsoil structure, and in particular on the local S-wave velocity profile.

The H/V curve is obtained by single station microtremor recordings. Similarly to the RWDC, also the H/V curves are related to the presence of surface waves in the seismic noise, and in particular to the particle motion, also known as ellipticity, of Rayleigh waves. Therefore, also the H/V provides information on the subsoil structure of a site. Specifically, H/V curves are strongly conditioned by the properties (depth and S-wave velocity contrast) of the interface between the soft sediment and the bedrock. Parolai *et al*. [34,35] confirmed that seismic noise H/V curves exhibit a good agreement with the H/V curves obtained from earthquake recordings, especially with regards to the value of the fundamental resonance frequency of the sedimentary cover, and hence provides some indication of the sedimentary cover thickness.

Various processing techniques for estimating the Rayleigh and Love wave phase velocities have been proposed [e.g., see 4 for a complete review of methods]. Because of its relative simplicity, at this stage of the system’s development, the traditional Extended Spatial AutoCorrelation (ESAC [1,3,4]) method using only the vertical component of ground motion is used for the estimation of the Rayleigh wave dispersion curve. A number of studies [e.g., 3,7,30] have shown that by using RWDC, the characterization of the local *S*-wave velocity profile can be obtained with a good accuracy, especially when a priori information about the total sedimentary cover thickness is available in advance.

We followed the procedure described by [7] in order to compute the RWDC with the ESAC procedure [3]. Practically, the procedure consists of estimating at each angular frequency *ω* the experimental, azimuthally averaged space-correlation values *ϕ*(*ω*) for every pair of stations by means of [3,4]:

(1) ϕjn(ω)=1M∑m=1MRe(Smjn(ω))1M∑m=1MSmjj(ω)∑m=1MSmnn(ω)

where *_m_S_jn_* is the cross-spectrum for the *m*th segment of data, between the *j*th and the *n*th stations and *M* is the total number of used segments. The power spectra of the *m*th segments at station *j* and station *n* are *_m_*S*_jj_* and *_m_*S*_nn_*, respectively. The phase velocity *c(ω*_0_*)* at each frequency is then estimated by a fitting procedure (*i.e.*, an iterative grid-search procedure) between the experimental spatial correlation values for all the possible station pairs plotted as a function of distance *r*, and theoretical values estimated by a Bessel function of the form:

(2) $$\phi \left(r,\omega_{o}\right)=J_{0}\left(\frac{\omega_{0}}{c\left(\omega_{0}\right)}r\right)$$

The tentative phase velocity *c(ω*_0_*)* is generally varied over large intervals (e.g., between 100 and 3,000 m/s) in small steps (e.g.1 m/s). The best fit is achieved by minimizing the root mean square (RMS) of the differences between the values calculated using equations (1) and (2). Data points that differ by more than two standard deviations from the value obtained with the minimum-misfit velocity can be removed before the next iteration of the grid-search [7].

Concerning the H/V curve, Nogoshi and Igarashi [36] suggested the normalization of the horizontal spectral amplitude with respect to the vertical one, with the aim of minimizing the source function effects in the noise spectra. In practice, the method for evaluating the H/V curves consists simply of (1) merging the two Fourier spectra of the two horizontal components, X*(ω)* and Y*(ω)*, of motion to obtain a single combined horizontal, H*(ω)*, component, and (2) computing the ratio between the H*(ω)* spectra with the one from the vertical component. We compute the modulus of the H*(ω)* combined spectra by the root mean square average (RMS):

(3) $$H_{\left(\omega \right)}=\sqrt{\left(X_{\left(\omega \right)}^{2}+Y_{\left(\omega \right)}^{2}\right)/2}$$

As discussed before, both the RWDC and H/V from the surface-wave analysis provides the necessary information for the S-wave velocity estimation. However, the relationship between the Rayleigh wave velocities and the H/V curve with the S-wave velocity and thickness of sediments is not linear. Therefore, analyses conducted to determine the subsoil S-wave velocity profile from surface-wave curves consists of solving a non-linear inverse problem. Unfortunately, non-linear inverse methods are generally time consuming, and thus not suitable for fieldwork uses. However, linearized procedures [37] might be used in the case some *a priori* constraints are available, or assumptions can be made about the S-wave velocity *versus* depth (*i.e.*, a realistic ‘input model’ can be defined). For sake of simplicity, in this study we used this approach, which can be especially useful for real-time S-wave velocity estimates. In particular, the linearized inverse problem of surface-wave curves (*i.e.*, RWDC or H/V) is solved using Singular Value Decomposition [SVD, 38] and by the generalized least-squares iterative minimization of the RMS of differences between observed and theoretical curves. Because of the non-linearity of the problem, the inversion is repeated, (1) until the RMS ceases to change significantly, and (2) starting from different input models.

This inversion approach is particularly suitable for preliminary analysis during the field measurements, since it has the advantage of being computationally efficient, and thus, easy to implement in real-time for retrieving S-wave estimates. Of course, in the event that other information concerning the subsoil structure is not available, and the S-wave velocity structure is particularly complicated, the preliminary field model from the linearized inversion procedure should be validated by post-acquisition robust, but also time-consuming, non-linear inversion analysis [6,39].

### 4.2. Data Recording, Analysis and Results

**Figure 5.**
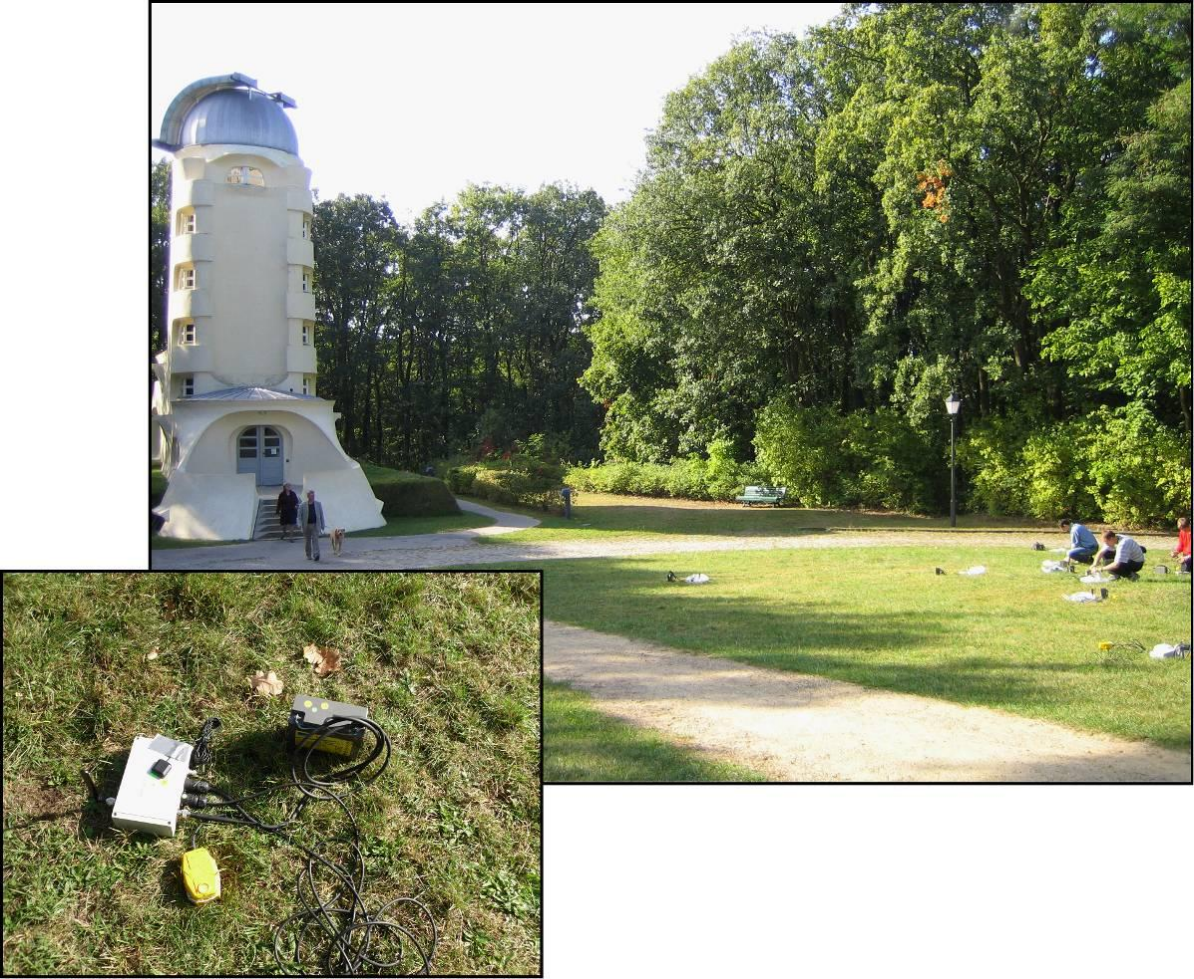
Field test with the GFZ-WISE system at the Alfred Einstein Science Park in Potsdam, Germany. In the *inner inset* it is shown an example of GFZ-WSU installation.

On the 6th of October 2009, the first field test with the GFZ-WISE system was performed at the Alfred Einstein Science Park in Potsdam, Germany (Figure 5).

The geology of Brandenburg, the region where the site is located, is representative for large areas of northern Germany, being characterized by Quaternary sediments formed during the last glacial period (Weichsel), overlying Tertiary clays. In particular, at the investigated site the Quaternary sediments are mainly fluvial and glacial sands [40].

**Figure 6.**
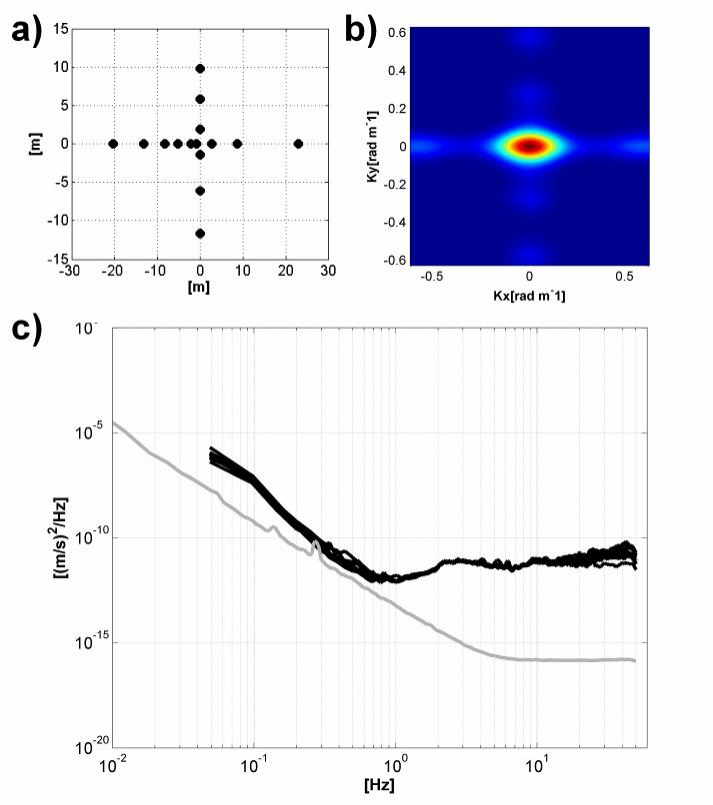
(a) Array geometry. (b) array response function. (c) experimental (*black lines*) and self-noise (*gray lines*) WSU spectra.

The array consisted of 15 GFZ-WSUs (Figure 5 and Figure 6) equipped with standard 3D SM-6/B 4.5 Hz external geophones that were installed so as to obtain a good coupling between the instrument and soil (Figure 5, inner inset). We selected a simple cross-shaped 2D geometry (Figure 6a), and ambient seismic noise was recorded at a sampling rate of 100 Hz for about 1 hour, which guarantees the statistical stabilization of the signal [41].

The energy required by the GFZ-WSUs was provided by 17 Ah batteries. The maximum inter-station distance, which controls the array resolution, and the minimum one which constrains which wavelengths will be affected by spatial aliasing, were 43 and 1.5 meters, respectively. Despite the selected geometry being quite simple, the array response function (*i.e.*, the transfer function of the array, which depends only on the distribution of stations in the array, and controls the spatial accuracy and resolution of signals that can be recorded by that array) computed for the frequency 10 Hz [42] does not show for the range of wave numbers of interest other peaks that would cause aliasing than the central one (Figure 6b).

The seismic noise data were recorded contemporary in real-time by two different operators, which established a WLAN-connection with two different GFZ-WSUs of the network, and were running the SeisComP software on their laptops. The observed delays during the multi-hops transmission of data from all the stations were constantly less than two seconds.

Before the analysis, all recordings were corrected for the instrumental response, considering the calibration parameters of each sensor. Then, the spectra of the recorded signals were compared with the self-noise spectra in order to identify the usable frequency range in the analysis. It is worth to point out that when the amplitudes of seismic noise at certain frequency become smaller than the self-noise (*i.e.*, also known as internal noise) of the instrument, the recorded signals do not provide anymore information about the propagation of waves into the ground. Hence, they must be discharged from the analysis. Of course, since the self-noise is a steady characteristic of the instrument, while the amplitude of the ambient noise changes with time, the comparison of the experimental spectra with the self-noise one should be always performed during each survey. Figure 6c shows that the experimental spectra tend to approach the self-noise for frequencies lower than 1Hz. Therefore, we limited the RWDC and H/V analysis to frequencies higher than this 1 Hz threshold.

The RWDC was computed using data from all stations of the array, following the procedure described previously. We used non-overlapping time windows 30 seconds long and tapered with a 5 per cent cosine function before the computation of the spectra, extracted from the 1 hour seismic noise recording signal.

**Figure 7.**
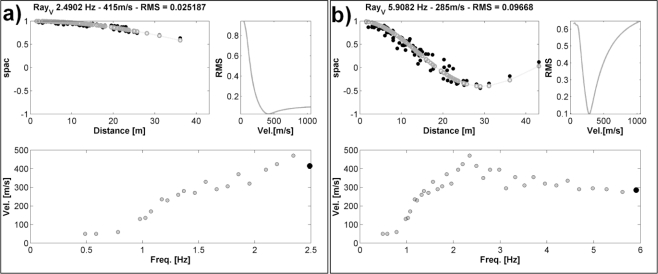
Real-time fitting procedure for Rayleigh-wave velocity estimation. (a) Analysis for the frequency 2.49 Hz. In the *upper-right panel* is shown the comparison between the observed spatial correlation function (*black dots*) and the theoretical Bessel function values for the best-fit velocities (*gray dots and line*). In the *upper-left panel* is shown the RMS for the tested velocities. In the *lower panels* are shown the evolutionary, real-time dispersion curve estimations, where the Rayleigh-wave velocities for the actual (*black dot*) and previous (*gray dots*) analyzed frequencies are shown. (b) same as (a) but for the frequency 5.9 Hz.

Figure 7 provides a view of the real-time analysis performed for retrieving the RWDC. In particular, Figure 7a,b, upper panels, show snapshots of the theoretical Bessel function fitting procedure with the experimental spatial correlation values, when plotted as a function of distance *r*, together with the root mean square values for the velocity explored during the grid search procedure. The high quality of the fitting results indicates that reliable phase velocity estimates were retrieved. In fact, in the case the GFZ-WSUs were not perfectly synchronized and the very small amplitude frequencies higher than 1Hz properly recorded, it would have been impossible to obtain phase differences among all the couple of sensors in the array in a relation each other that allow estimating the velocity of the Rayleigh waves transmitting throughout the array.

Moreover, Figure 7a,b, lower panels, provide an example of the phase velocity values estimated during the real-time analysis. A real-time analysis of the data allows the operator to observe in real time both the quality of the space-correlation values fitting procedure, and the evolution of velocity estimates with the increasing of the frequency.

**Figure 8.**
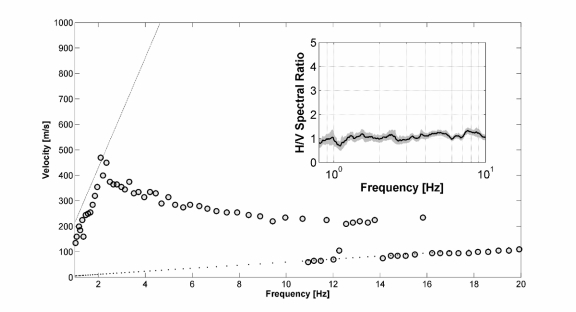
Results of the surface-wave analysis. *Main panel*: Rayleigh-wave dispersion curves. Experimental phase velocities (*gray dots*), theoretical limit for the spatial aliasing (*dotted line*), and theoretical limit for the spatial resolution (*black line*). *Inset:* horizontal-to-vertical spectral ratio curve. Average H/V values (*black line*), and the 95% confidence interval (*gray area*).

Figure 8 shows the final result of the ESAC analysis together with theoretical limits for the spatial aliasing and spatial resolution estimated from the array response function [42]. The RWDC is characterized by a quite regular increase in the Rayleigh wave phase velocities from about 15 Hz down to 2.5 Hz, which indicates a regular and smooth increase of the S-wave velocities with depth in the subsoil. It is worth noting that the limits of the dispersion curve at both high and low frequencies are very well correlated with the predicted resolution and spatial aliasing constraints. That is, at low frequencies the phase velocities can be estimated until 2.5 Hz, which is in agreement with the resolution boundary estimated considering the maximum inter-station distance [4]. Similarly, the linear trend of velocities increasing with frequency from about 10 Hz onwards is clearly related to the spatial aliasing [4].

The H/V spectral ratios [43] were computed using for all stations the same time-window length adopted for the estimation of the dispersion curve. The Fourier spectra were computed for each noise component and smoothed using a Hanning window of 10% relative bandwidth. This ensures the reduction of numerical instabilities while preserving the major features of the spectra. The resulting spectral ordinates relative to the horizontal components were geometrically averaged and divided by the vertical spectral ordinate to compute the H/V function. Finally, H/V ratios obtained by considering the resultant time windows were then averaged to compute the final H/V curves along with the relevant 95% confidence interval. Figure 8, inner inset, shows the example of one estimated H/V. In agreement with the results about the subsoil structure provided by the RWDC, the H/V is flat and indicates that down to the first few tens of meters depth under the site there are no high-impedance contrast boundaries. A large impedance contrast might be expected at greater depths, requiring the identification of a peak in the H/V ratio curve at frequencies well below those have to be exploited from data recorded by the GFZ-WSU.

**Figure 9.**
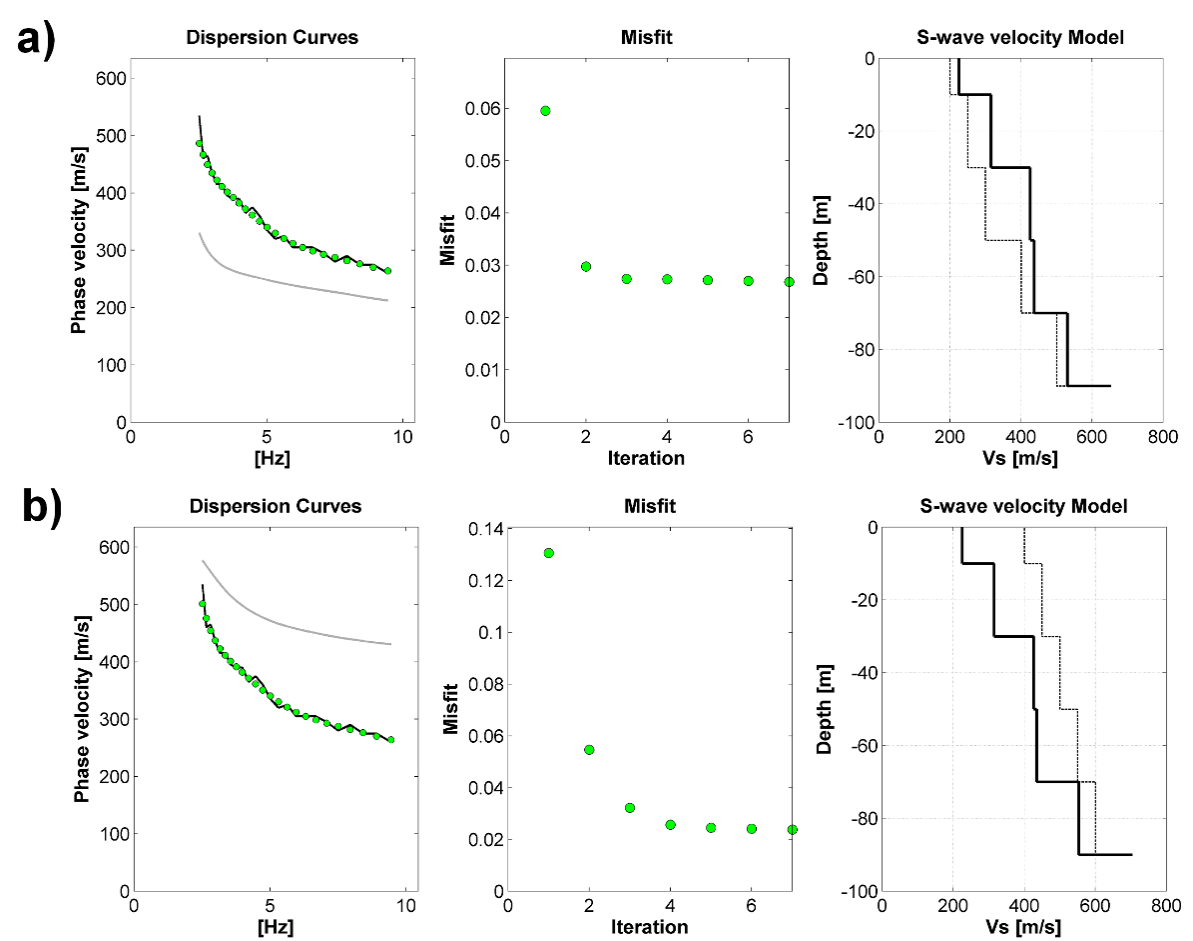
Results of the inversion analysis starting from different input models. (*a*) Under-estimated input model. In the *left-panel* are shown the experimental phase velocities (*black line*), those for the best-fit model (*green dots*), and the dispersion curves for the input model (*gray line*). In the middle-panel is shown the misfit for each iteration (*green dots*). In the *right-panel* are shown the S-wave velocities for the input (*thin line*) and final best-fit (*thick line*) models. (*b*) same as (*a*), but for an over-estimated input model.

Finally, the inversion procedure was carried out in order to retrieve the S-wave velocity profile. Considering the general information about the subsoil structure that can be obtained by a visual inspection of both the H/V and RWDC, and the fact that the H/V is flat, only a linearized inversion of the RWDC for a frequency range between about 2.5 Hz and about 10 Hz, was performed (Figure 9).

In general, for simple S-wave velocity structures, a reasonable input model for the inversion procedure can be obtained by considering the simple rules of assigning 110% of the velocity at a certain frequency to a depth equal to half the wavelength (evaluated as velocity/frequency) of the corresponding frequency [4].

However, in order to verify the robustness of the inversion procedure, we performed the iterative inversion starting from two extreme velocity profiles (Figure 9). That is, we started the inversion from input models that definitely under- and over-estimate the real S-wave velocity profile. It is worth to note that, independent of the starting model, after only few inversion steps, there is a convergence towards almost the same velocity model, which is able to fully justify the observed data. Therefore, even if the good fit of data cannot be considered always as an absolute measure for the high quality of the inverted model, we think that the estimated S-wave velocity model is a reliable representation of the subsoil structure down to about 90 meters (Figure 9).

Unfortunately, *a priori* information on the S-wave velocity at the site was not available. Thus, a direct comparison of the estimated S-wave velocity profile with an alternative one was not possible. However, the tests we are carrying out with the GFZ-WISE aim primarily to verify the methodological aspects, as the communication efficiency between the sensors and a user’s laptop for the data retrieval, and the station performance while recording real seismic noise. Nevertheless, the obtained S-wave velocities are compatible with those observed for the same kind of sediments by Richwalski *et al*. [32].

## 5. Conclusions

We have presented a new system, GFZ-WISE, for performing dense 2D seismic ambient-noise array measurements. The system is made up of novel low-cost wireless sensing units (GFZ-WSU) designed to form dense wireless mesh networks (WMN). The GFZ-WSUs employ advances in various technologies to incorporate off-the-shelf sensors, processing and communications components into low-cost seismic sensing units that are linked by advanced, robust and rapid communications routing and network organizational protocols appropriate for WMNs. The reduced cost of the instruments (*i.e.*, less than one tenth of a standard instrument) and the possibility of creating dense, self-organizing seismic monitoring networks are key attributes that all new approaches to seismic noise surveys to be followed (e.g., within Mega-city and landslides monitoring).

Each of the GFZ-WSUs is able to collect, store and undertake preliminary analysis of data when only parameters (e.g., the horizontal-to-vertical curve of a site) are of concern. In addition, the stations create a seismic WMN, through which raw data and computed parameters can be communicated to a user’s external laptop running the SeisComP software, which is connected to any node that belongs to the network, allowing a user to perform real-time quality control, and analysis of seismic data. Furthermore, the self-organizing character of the network guarantees the functionality of the network even when some of the sensing units malfunction or are not directly in line-of-sight with the operators. This latter characteristic makes the GFZ-WISE system particularly attractive during survey in urban contexts for microzonation studies, when obstacles as buildings might constraint the geometry of the array for wireless systems centralized or standard cable-dependent systems. Further, during 2D array seismic noise measurements, which generally involve period of recording of few hours, the necessary energy can be provided by small 17 Ah batteries. Finally, thanks to the reduced dimension and weight of the stations, the system is easy to install.

Future applications of the GFZ-WISE system will include the monitoring of seismic noise, but when of interest also ground motion or other parameters, for urban sites in earthquake prone areas and landslides. For such long-term monitoring applications, the necessary energy for the GFZ-WSUs should be provided by a buffer battery (e.g., 40 Ah) connected to a solar panel (e.g., 60 W) through a solar controller. In urban context, the reduced cost of the GFZ-WSUs would enable to create dense network for the characterization and real-time monitoring of the variations of the subsoil mechanical properties following the shaking of an earthquakes, as well as to provide new observations concerning the soil-structure interaction effects. However, it worth to specify that for seismological purposes (e.g., studies of the seismic sources, or basins with sedimentary cover thickness of several hundred of meters) where the target signals interest frequencies below 1 Hz, the GFZ-WSUs should be equipped by passive velocimeter sensors with lower corner frequency and higher resolution, but higher cost, than those of the sensor used in this study. Similarly, for strong motion studies, when input signals have very large amplitude, the MEMS accelerometers should be used.

For monitoring application of special sites for which early warning or rapid response actions would also required, as landslides, special nodes that incorporate additional communications hardware, e.g., Internet connection, satellite phones, VSAT, *etc*., may serve as entities able to communicate data and parameters to outside of the network, such as a disaster management center. In particular, in the future it is intended to use the GFZ-WISE at landslide sites to monitor and analyze a combination of parameters (e.g., the S-wave velocity, ground motion, groundwater level variation, and rain gauge), and together with dedicated decision making algorithms, to detect and locate changes within the landslide. Hence, the GFZ-WISE system might provide real time early warning information about the possible landslide activation after earthquakes or meteorological events.

Results of the tests performed indicate an excellent performance of the innovative instruments when used in seismic site effect surveys. In fact, the GFZ-WSU displayed for frequencies higher than 1 Hz a performance comparable to other standard, high sensitivity and higher costs seismic stations. Moreover, during a field experiment, the GFZ-WISE was found to be effective in providing to external users real-time access to the data. Therefore, results shown in this study indicate that in the near future, dense arrays of low-cost wireless sensors might be successfully and profitably deployed for the purpose of site-effects studies and monitoring activities, providing a worthwhile contribution to the reduction of seismic risk in urban areas.

## Figures and Tables

**Table 1.** Technical specifications of the low-cost Wireless Sensing Units.

**Technical Data:**	
Size	200 × 150 × 80 mm^3^
Weight	1.1 kg
Power Consumption	∼5 W@12 V
2 × WLAN	2.4 GHz / 5.7 GHz /LAN (opt.)
Power Supply	10...15V DC
ADC	4 × ADC (24Bit)
Sensors	Ext. Geophones / internal 3 Axis MEMS-Accelerometer GPS Receiver & external GPS Antenna Input

**Table 2.** Technical specifications of the various components that make up the ALIX board as currently used in the Wireless Sensing Units.

**ALIX board**	
CPU	AMD L×800 (500 MHz)
DRAM (dynamic random access memory)	256 MB SDRAM (synchronous dynamic random access memory)
Operating system	OpenWRT
Storage	Compact Flash card, currently 2 GB
Power consumption	3 to 5 W at 12 V DC (excluding miniPCI cards)
Safety features	Watchdog timer built into the CPU, LM77 thermal monitors
User interface	Three front leds, console I/O redirected to serial port
Possible expansions	LPC bus for adding more serial ports, ISA style I/O, GPIO and I^2^C bus
Connectivity	One Ethernet channel (National DP83816), two miniPCI slots, one serial port
BIOS	tinyBIOS version 1.11 LAN (10/100) 2 × USB

**Table 3.** Technical specifications of the various components that make up the ADC board as currently used in the Wireless Sensing Units.

**ADC Board (GFZ)**	
Number of channels	Four (eight Ch. Opt.)
AD convertor resolution/effective resolution	24 Bit, effective 19 Bit (20 Bit in HR Mode)
Input voltage range	+/− 2.5 V
Input impedance	10 kΩ
Bit weight	0.3 μV (30 nV with preamp of gain 10)
Sample rate	Sample Rate:100 sps (200 & 400 sps not yet by software supported)
Signal bandwidth (−3dB)	50 Hz/fs/2
Stop bandwidth attenuation	>100 dB
Analogue anti-alias filter	
Timing	Onboard GPS Receiver for Timing & Position Data
Timing accuracy	Time Base better 2.5 ppm
Digital output	USB (1 × virtual com-port, 115 kBaud data/GPS)
Temperature range	−20° to +70 °C
Power supply	9 to 18 V (on board DC/DC converter) or +5 V (USB)
Power consumption	540 mW (low power mode) / 720 mW (high resolution mode)
*Different Modules available*	
**MODULES**	
**MEMS Accelerometer**	3 Axis, +/−1.7 g
	Resolution ∼0.2 mg (rms)
	Tilt Resolution ∼ 0.01° (Temp. Correction required)
**Preamplifier**	4 Channels
	Gain × 10 (default, set by R)
	Noise ∼80 nV(rms) @100 sps
**Geophones**	3D SM-6/B 4.5 Hz, or any other passive geophone

## Acronyms:

2D: two dimensional
1D: one dimensional
ADC: Analog-to-Digital Converter board
ESAC: Extended Spatial AutoCorrelation
GFZ-WISE: GeoForschungsZentrum WIreless SEismic array
GFZ-WSU: GeoForschungsZentrum Wireless Seismic Unit
H/V: Horizontal-to-Vertical spectral ratio curve
MEMS: Micro Electro Mechanical Systems
MPR: MultiPoint Relays
RMS: Root Mean Square
RWDC: Rayleigh Wave Dispersion Curve
S-wave: shear waves
WRAP: Wireless Router Application Platform
WMN: Wireless Mesh Network
